# The association between social network index, atrial fibrillation, and mortality in the Framingham Heart Study

**DOI:** 10.1038/s41598-022-07850-9

**Published:** 2022-03-10

**Authors:** Jelena Kornej, Darae Ko, Honghuang Lin, Joanne M. Murabito, Emelia J. Benjamin, Ludovic Trinquart, Sarah R. Preis

**Affiliations:** 1 National Heart, Lung, and Blood Institutes Framingham Heart Study, Framingham, MA USA; 2 Section of Cardiovascular Medicine, Boston Medical Center, Boston University School of Medicine, 72 E Concord St, Boston, MA 02118 USA; 3 Section of Computational Biomedicine, Department of Medicine, Boston University School of Medicine, Boston, MA USA; 4 Section of General Internal Medicine, Department of Medicine, Boston Medical Center, Boston University School of Medicine, Boston, MA USA; 5 Department of Epidemiology, Boston University School of Public Health, Boston, MA USA; 6 Department of Biostatistics, Boston University School of Public Health, Boston, MA USA

**Keywords:** Cardiology, Health care, Medical research, Risk factors

## Abstract

Social isolation might be considered as a marker of poor health and higher mortality. The aim of our analysis was to assess the association of social network index (SNI) with incident AF and death. We selected participants aged ≥ 55 years without prevalent AF from the Framingham Heart Study. We evaluated the association between social isolation measured by the Berkman-Syme Social Network Index (SNI), incident AF, and mortality without diagnosed AF. We assessed the risk factor-adjusted associations between SNI (the sum of 4 components: marriage status, close friends/relatives, religious service attendance, social group participation), incident AF, and mortality without AF by using Fine-Gray competing risk regression models. We secondarily examined the outcome of all-cause mortality. We included 3454 participants (mean age 67 ± 10 years, 58% female). During 11.8 ± 5.2 mean years of follow-up, there were 686 incident AF cases and 965 mortality without AF events. Individuals with fewer connections had lower rates of incident AF (*P* = 0.04) but higher rates of mortality without AF (*P* = 0.03). Among SNI components, only social group participation was associated with higher incident AF (subdistribution hazards ratio [sHR] 1.35, 95% CI 1.16–1.57, *P* = 0.0001). For mortality without AF, social group participation (sHR = 0.81, 95% CI 0.71–0.93, *P* = 0.002) and regular religious service attendance sHR = 0.76, 95% CI 0.67–0.87, *P* < 0.0001) were associated with lower risk of death. Social isolation was associated with a higher rate of mortality without diagnosed AF. In contrast to our hypothesis, we observed that poor social connectedness was associated with a lower rate of incident AF. This finding should be interpreted cautiously since there were very few participants in the lowest social connectedness group. Additionally, the seemingly protective effect of social isolation on AF incidence may be simply an artifact of the strong association between social isolation and increased mortality rate in combination with the large number of deaths as compared to AF events in our study. Further study is warranted.

## Introduction

Social isolation plays a critical role in the determination of health status and is associated with higher risk of mortality^1,2^. A person’s level of social integration is characterized by factors such as the presence of close personal ties to family and friends and social ties to community. Low social integration is associated with both cardiovascular disease (CVD) incidence and mortality^3–5^. Social isolation might be considered as a marker of poor health and worse prognosis showing up to 75% and 62% higher risk of mortality in women and men, respectively^5^. However, the results are inconsistent^6^.

Social isolation is usually associated with an age-dependent traumatic situations such as loss of partner (widowhood, divorce)^7^ or a decline in functional capacity^5^. Based on a cross-sectional analysis of the Women's Health Study, traumatic life events were associated with higher odds of prevalent AF^8^. There is evidence that psychosocial stress and associated negative emotions are common triggering factors for AF paroxysms^9^.

The Berkman-Syme Social Network Index (SNI) is a self-reported measure of social ties based on an individual’s number of social ties^1^. As demonstrated by Berkman et al., individuals without emotional support (ascertained before myocardial infarction) had twofold increased risk of death in-hospital and 6-month post-myocardial infarction^10^.

The role of social integration/isolation on AF incidence and mortality in the community is incompletely understood. We hypothesized that low social support is associated with increased risk of incident AF, mortality without diagnosed AF, and all-cause death.

## Methods

### Study sample

The Framingham Heart Study is a multi-generational prospective cohort study. Starting from October 1948, 5209 participants who were residents of Framingham, MA were enrolled in the Original Cohort of Framingham Heart Study^11^. Enrollment of the Offspring Cohort began in 1971 with inclusion of 5124 children (and their spouses) of the Original Cohort participants^12^. In 1994, the Framingham Heart Study’s Omni 1 Cohort began and included 507 African-American, Hispanic, Asian, Indian, Pacific Islander, and Native American participants from Framingham and surrounding towns. The Original Cohort participants underwent biennial research examinations, whereas the Offspring and Omni 1 cohorts were seen every 4 to 8 years.

For the present analysis, we included participants who were aged ≥ 55 years when they attended the examination cycle at which the Berkman SNI questionnaire was administered. The Berkman SNI questionnaire was given at the following exams: Original cohort exams 25 (1997–1999) to 31 (2010–2011), Offspring cohort exam 7 (1998–2001), Omni 1 cohort exam 2 (1999–2001), and at a call-back examination ~ 3 months after Offspring exam 8 (2005–2008) and Omni 1 cohort exam 3 (2007–2008) for participants in an ancillary study of brain magnetic resonance imaging (MRI) and dementia. For all participants, we selected their first SNI measured at age ≥ 55 years.

All procedures performed in studies involving human participants were in accordance with the ethical standards of the institutional and/or national research committee and with the 1964 Helsinki Declaration and its later amendments or comparable ethical standards. The Framingham Heart Study protocol was approved by the Boston University Medical Center Institutional Review Board (Approval Number H-32132) and all participants (or proxies) signed informed consent.

### Exposure assessment

Social integration and isolation were assessed based on the Berkman-Syme SNI questionnaire as previously described^1,13^. The SNI index has been commonly used in prior research as a measure of social isolation^14,15^. The four SNI domains were scored as follows: currently married (no = 0; yes = 1); number of close friends and relatives (0–2 friends/relatives = 0; ≥ 3 close friends/relatives = 1); participation in a social group (no = 0; yes = 1); religious meeting or service attendance (attends a few times per year or less = 0; attends at least once or twice a month = 1). The latter two categories were mutually exclusive from each other. The sum of the four categories represents the SNI score: low (score of 0), medium–low (1), medium (2), medium–high (3), or high (4) socially connected categories. The SNI score was analyzed as an ordinal variable, with a score of 4 (high social connectedness) being the reference group.

### Atrial fibrillation ascertainment

Ascertainment of AF in the Framingham Heart Study has been described previously^16^. All participants undergo an electrocardiogram (ECG) as a routine part of their research exam. A diagnosis of AF was given if either AF or atrial flutter was observed on the ECG or if AF was documented in participants’ outside medical records, interim hospitalizations, outside ECGs, or Holter monitor results.

### Covariate assessment

For the present analysis, covariate measurements were taken from the same exam in which participants completed the SNI questionnaire. For the Offspring and Omni 1 participants who completed the SNI as part of the brain MRI ancillary study, covariates were measured at the most recent research exam within 1 year of their SNI measurement. The study participants underwent routine research examinations approximately every 2 (Original cohort) or 4 to 8 years (Offspring/Omni 1 cohort). At each visit, a physician obtained a medical history and administered a physical examination. Height, weight, smoking status, diabetes, blood pressure and antihypertensive medication use were recorded during each examination.

All Framingham Heart Study participants are routinely monitored for the development of any cardiovascular event and/or death. All events are adjudicated by a panel of 2–3 clinicians (the Framingham Endpoint Review Committee) using Framingham research center examinations and outside medical records or hospitalization charts. Heart failure was diagnosed based on the simultaneous presence of at least two major criteria OR one major criterion and two minor criteria as previously described^17^. History of myocardial infarction was designated if there were at least two of three findings: (1) symptoms indicative of ischemia; (2) changes in blood biomarkers of myocardial necrosis; (3) serial changes in the electrocardiograms. Deaths were documented by death certificates.

### Statistical methods

Descriptive statistics were calculated using means and standard deviations or frequency counts and percentages, as appropriate, for each level of SNI score. Participants were followed from the exam date of their SNI assessment until the occurrence of AF, death, loss to follow-up, or December 31st, 2016, whichever occurred first. We divided the outcome of all-cause mortality into two categories depending on whether the participant experienced an incident AF event over the course of follow-up prior to dying: (1) those who experienced an AF event prior to death (“post-AF mortality”) and (2) those who did not experience an AF event prior to death. The primary outcomes for the present analysis were incident AF and mortality without AF diagnosis while all-cause mortality was considered as a secondary outcome.

Cox proportional hazards models were used to calculate cause-specific hazard ratios (HR) and 95% confidence intervals (CI) for the association between SNI score and each outcome (incident AF, mortality without an AF diagnosis], and all-cause mortality). We analyzed the SNI score as a categorical variable, as was done in the original publication of the describing the index^1^. Indicator variables were used for each level of SNI score, with the highest score of 4 being the referent group. Models were also constructed for each of the individual SNI components. Model 1 was adjusted for age, sex, and time between SNI measurement and covariate measurement. Model 2 was further adjusted for height, weight, systolic blood pressure, diastolic blood pressure, hypertension treatment, diabetes, current smoking, history of myocardial infarction, and history of heart failure—the factors associated with AF in the CHARGE-AF model^18^. The proportional hazards assumption was verified using the Supremum Test for Proportional Hazards in the PHREG procedure in SAS. All variables satisfied the proportional hazards assumption.

For the incident AF outcome, Fine-Gray models, which account for the competing risk of mortality, were used to calculate subdistribution hazards ratios (sHR)^19^. Similarly, for the mortality without AF diagnosis outcome, the Fine-Gray models were used to account for the competing risk of AF. Results for both the cause-specific HRs and the subdistribution HRs are presented in the results, as has been previously recommended^20^.

To handle missingness of covariate data, the PROC MI procedure in SAS was used to implement multiple imputation with fully conditional specification. Approximately 19% of participants were missing diabetes status since fasting blood glucose was not measured in the Original cohort. For the remaining covariates, the percentage of missing ranged from 0 to 2% (Table S1). The imputation models were created separately for each of the three outcomes (incident AF, mortality without AF diagnosis, all-cause mortality). The following variables were included in the imputation models: age, sex, cohort, height, weight, systolic blood pressure, diastolic blood pressure, hypertension treatment, current smoking, fasting blood glucose, diabetes treatment, history of myocardial infarction, history of heart failure, SNI score, indicator for event occurrence, and the Nelson-Aalen estimate of cumulative hazard for each participant^21^. A total of 30 imputed datasets were created and the coefficients were combined using Rubin’s rule with the PROC MIANALYZE procedure in SAS. The significance of categorical predictors was tested using the median of the *p*-values from the overall significance tests of the imputed datasets^22^. All analyses were performed using SAS version 9.4 (Cary, NC). A two-sided *p*-value of < 0.05 was considered statistically significant.

## Results

### Study sample characteristics

**Figure 1:**
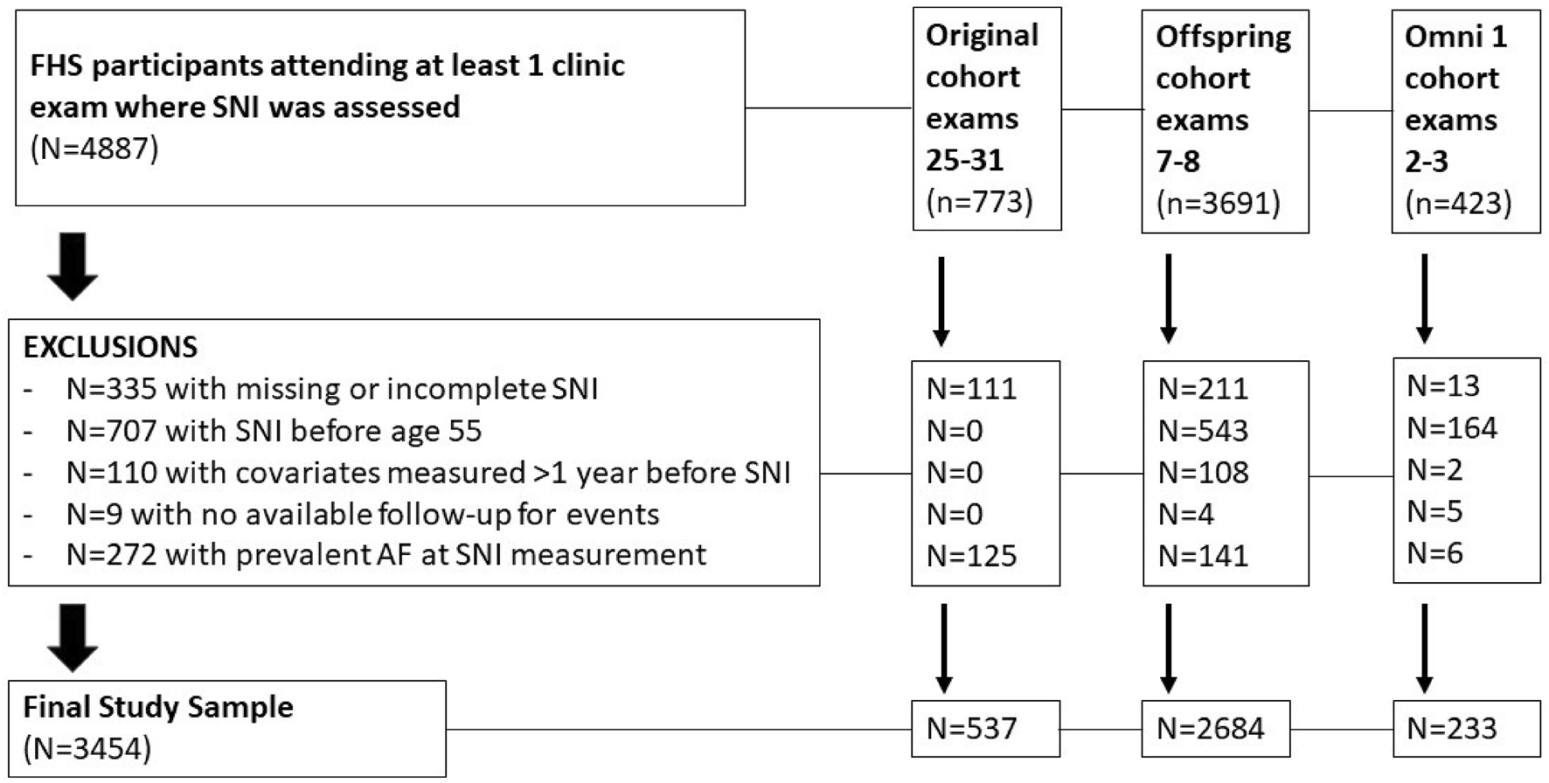
Study sample selection flow chart.

The study sample selection is shown in Fig. 1. A total of 4887 participants attended at least one exam during which SNI was assessed. We excluded participants with missing or incomplete SNI questionnaires (*n* = 335), SNI assessment available only prior to age 55 years (*n* = 707), no covariate measures within one year of SNI assessment (*n* = 110), lack of AF follow-up time after SNI assessment (*n* = 9), and prevalent AF at the time of SNI assessment (*n* = 272).

The present analysis included 3454 participants (mean age 67.4 ± 9.8 years, 57.7% females) from the Framingham Heart Study Original (*n* = 537), Offspring (*n* = 2684) and Omni 1 (*n* = 233) cohorts. During a mean (± SD) follow-up of 11.8 ± 5.2 years, there were 686 incident AF events and 1373 total deaths. There were 965 deaths among participants who remained free of AF diagnosis during the follow-up period.

Clinical characteristics of the study sample, stratified by the Berkman-Syme social network index (SNI) score, are presented in Table 1. A total of 81 (2.3%) participants had a SNI score of zero (“low” social connectivity), 525 (15.2%) had a score of 1 (“medium–low”), 1119 (32.4%) had a score of 2 (“medium”), 1079 (31.2%) had a score of 3 (“medium–high”), and 650 (18.8%) had a score of 4 (“high” social connectivity). As compared to participants in the high SNI group, those with low SNI were slightly older, more likely to be female, smoke currently, belong to the Original cohort, and were less likely to belong to the Omni 1 cohort. Clinical characteristics for each cohort separately are presented in Supplemental Tables S1-3.

**Table 1:** Study sample characteristics.

	Social network index score					Total (*N* = 3454)
	Low (0) (*N* = 81)	Medium–low (1) (*N* = 525)	Medium (2) (*N* = 1119)	Medium–high (3) (*N* = 1079)	High (4) (*N* = 650)	
Age, years	68.7 ± 12.0	68.1 ± 11.1	67.4 ± 10.0	67.6 ± 9.6	66.4 ± 8.2	67.4 ± 9.8
Female sex	54 (66.7)	297 (56.6)	605 (54.1)	670 (62.1)	367 (56.5)	1993 (57.7)
Years of follow-up for incident AF	10.0 ± 5.2	11.1 ± 5.5	11.6 ± 5.2	12.1 ± 5.0	12.4 ± 5.0	11.8 ± 5.2
Framingham heart study cohort						
Original	21 (25.9)	109 (20.8)	183 (16.4)	168 (15.6)	56 (8.6)	537 (15.6)
Offspring	60 (74.1)	397 (75.6)	885 (79.1)	826 (76.6)	516 (79.4)	2684 (77.7)
Omni 1	0 (0.0)	19 (3.6)	51 (3.6)	85 (7.9)	78 (12.0)	233 (6.8)
Systolic blood pressure, mm Hg	130 ± 19	131 ± 20	133 ± 20	131 ± 19	130 ± 19	132 ± 19
Diastolic blood pressure, mm Hg	73 ± 10	73 ± 11	74 ± 10	73 ± 10	74 ± 10	73 ± 10
Height, inches	65 ± 4	65 ± 4	65 ± 4	65 ± 4	65 ± 4	65 ± 4
Weight, pounds	171 ± 51	167 ± 39	170 ± 38	168 ± 38	170 ± 36	169 ± 38
Hypertension treatment	33 (41.3)	234 (44.7)	487 (43.7)	439 (40.8)	261 (40.2)	1454 (42.3)
Current smoking	25 (30.9)	91 (17.4)	126 (11.3)	84 (7.8)	30 (4.6)	356 (10.3)
Diabetes	8 (14.3)	45 (11.4)	121 (13.4)	108 (12.3)	71 (12.4)	353 (12.6)
History of heart failure	1 (1.2)	13 (2.5)	12 (1.1)	10 (0.9)	5 (0.8)	41 (1.2)
History of myocardial infarction	2 (2.5)	23 (4.4)	52 (4.7)	34 (3.2)	19 (2.9)	130 (3.8)
Social network index components						
Currently married	0 (0.0)	190 (36.2)	743 (66.4)	755 (70.0)	650 (100.0)	2338 (67.7)
> 2 close friends and > 2 close relatives	0 (0.0)	272 (51.8)	912 (81.5)	981 (90.9)	650 (100.0)	2815 (81.5)
Regular religious service attendance	0 (0.0)	33 (6.3)	312 (27.9)	807 (74.8)	650 (100.0)	1802 (52.2)
Participates in social group	0 (0.0)	30 (5.7)	271 (24.2)	694 (64.3)	650 (100.0)	1645 (47.6)

Tables values represent mean ± SD or *n* (%).

Prior to imputation, a total of 665 participants were missing one or more covariates. Details of the missingness are presented in Supplemental Tables S4 and S5. The most frequently observed missing variable was diabetes status (18.9% missing), since fasting blood glucose was not measured in the Original cohort, followed by height (2.4% missing). Compared to the complete cases, individuals with at least one missing covariate were significantly older, had higher systolic blood pressure and had lower SNI, and were more likely to be female and have a history of myocardial infarction and heart failure.

### Association between social network index and incident AF

**Figure 2:**
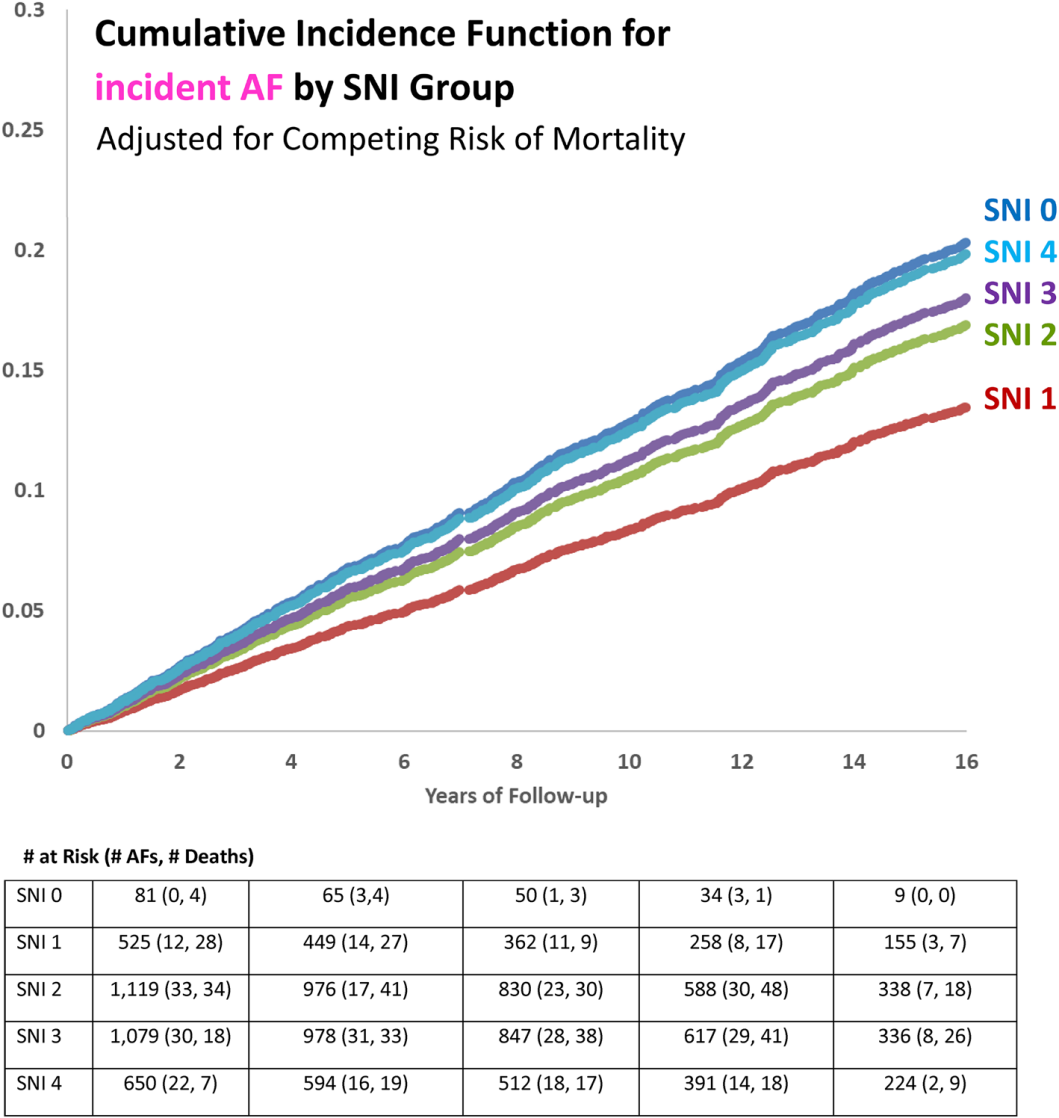
Association between SNI and incident AF.

Table 2 and Fig. 2 show the results for the association between SNI and AF. In both the age- and sex-adjusted and multivariable-adjusted Cox models, there was no evidence of association between SNI and incident AF. However, in the multivariable-adjusted Fine-Gray subdistribution hazards model, which was adjusted for the competing risk of mortality, there was evidence of association between SNI group and incident AF (*p*-value = 0.04). Compared to the high SNI group, participants with a medium–low SNI score had a lower incidence of AF (sHR = 0.66, 95% CI 0.50–0.87). There was no evidence of difference in AF incidence between the low, medium, and medium–high SNI groups as compared to the highest SNI group.

**Table 2:** Hazards ratios for the association between social network index and incident atrial fibrillation in the Framingham Heart Study Original, Offspring, and Omni 1 cohorts.

Social network index	# AF cases/# participants	Cox proportional hazards model				Fine-gray subdistribution hazards model	
		Model 1: Age- and sex- adjusted		Model 2: Multivariable-adjusted*		Model 3: Multivariable-adjusted* + adjustment for competing risk of mortality	
		HR (95% CI)	*P*-value	HR (95% CI)	*P*-value	sHR (95% CI)	*P*-value
**SNI score**							
0—Low	19/81	1.32 (0.82–2.14)	0.26	1.33 (0.81–2.16)	0.26	1.04 (0.64–1.69)	0.87
1—Medium–Low	86/525	0.80 (0.61–1.05)	0.10	0.78 (0.60–1.03)	0.08	0.66 (0.50–0.87)	0.003
2—Medium	224/1119	0.92 (0.74–1.14)	0.43	0.89 (0.72–1.11)	0.30	0.84 (0.67–1.04)	0.11
3—Medium–High	219/1079	0.93 (0.75–1.15)	0.48	0.92 (0.74–1.14)	0.44	0.90 (0.72–1.12)	0.33
4—High	138/650	1.00 (referent)	–	1.00 (referent)	–	1.00 (referent)	–
Total	686/3454	Overall *p*-value	0.27	Overall *p*-value	0.21	Overall *p*-value	0.04
**SNI components**							
**Currently married**							
Yes	446/1892	0.92 (0.77–1.09)	0.32	0.94 (0.79–1.12)	0.49	0.95 (0.80–1.14)	0.60
No	240/1116	1.00 (referent)	–	1.00 (referent)	–	1.00 (referent)	–
**Number of close friends and relatives**							
≥ 3 friends and ≥ relatives	551/2815	0.93 (0.77–1.13)	0.47	0.95 (0.79–1.15)	0.63	0.97 (0.80–1.18)	0.78
0–2 friends and 0–2 relatives	135/639	1.00 (referent)	–	1.00 (referent)	–	1.00 (referent)	–
**Frequency of religious service attendance**							
≥ 1 time per month	385/1802	1.02 (0.87–1.18)	0.85	1.01 (0.87–1.18)	0.88	1.16 (1.00–1.35)	0.05
< 1 time per month	301/1652	1.00 (referent)	–	1.00 (referent)	–	1.00 (referent)	–
**Social group participation**							
Yes	361/1645	1.26 (1.08–1.47)	0.003	1.25 (1.08–1.46)	0.004	1.35 (1.16–1.57)	0.0001
No	325/1809	1.00 (referent)	–	1.00 (referent)	–	1.00 (referent)	–

SNI, social network index; AF, atrial fibrillation; HR, cause-specific hazards ratio; sHR, subdistribution hazards ratio; CI, confidence interval.

All models are stratified by cohort membership and adjusted for time between SNI measurement and covariate measurement. Multiple imputation was implemented to handle missing covariate data.

*Adjusted for age, sex, height, weight, systolic blood pressure, diastolic blood pressure, hypertension treatment, current smoking, diabetes, history of myocardial infarction, and history of heart failure.

Of the four individual SNI components, social group participation was associated with higher incidence of AF in all models. In the multivariable-adjusted Fine-Gray subdistribution hazards model comparing social group participants to non-participants, the sHR for AF was 1.35 (95% CI 1.16–1.57, *p* < 0.0001). Supplemental Table S6 shows the results for the complete case analysis.

### Association between social network index and mortality without AF diagnosis

**Figure 3:**
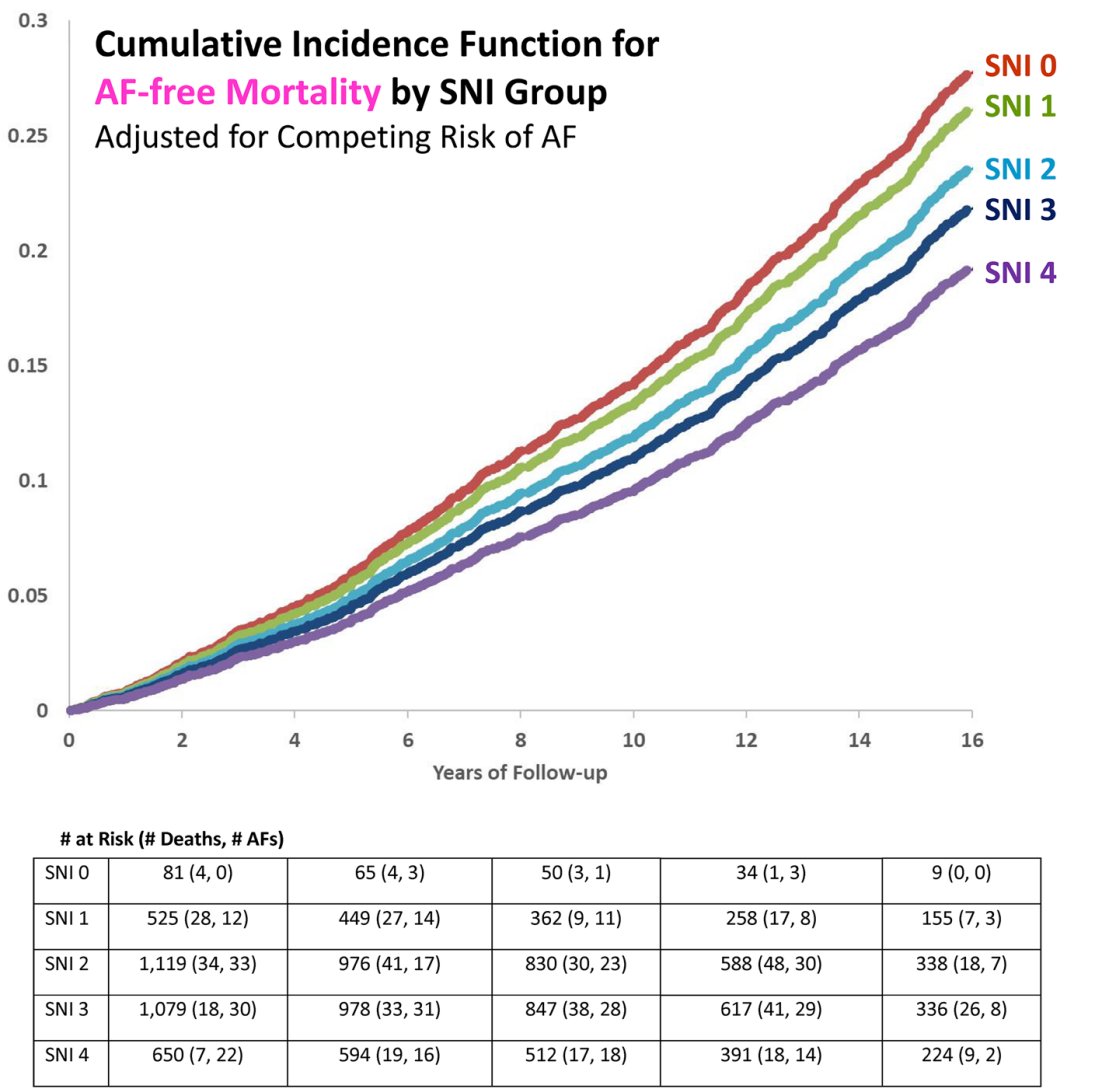
Association between SNI and mortality without AF diagnosis.

The results for the association between SNI and mortality without AF diagnosis are displayed in Table 3 and Fig. 3. In the age- and sex-adjusted Cox model, SNI group overall was associated with mortality without AF diagnosis (*p*-value = 0.0006). Participants in the lowest SNI group had 1.9 times the rate of death without AF diagnosis as compared to those in the highest SNI group (*p*-value = 0.002). The results for the multivariable-adjusted Cox model were similar but were slightly attenuated. In the multivariable-adjusted Fine-Gray subdistribution hazards model, which accounts for the competing risk of AF, the sHR for the lowest SNI group was attenuated and was no longer statistically significant (sHR = 1.50, *p*-value = 0.06). However, there was still an overall association for SNI score (*p*-value = 0.03).

**Table 3:** Hazards ratios for the association between social network index and incident mortality without AF diagnosis* in the Framingham Heart Study Original, Offspring, and Omni 1 cohorts.

Social network index	# Deaths/# participants	Cox proportional hazards model				Fine-gray subdistribution hazards model	
		Model 1: Age- and sex-adjusted		Model 2: Multivariable-adjusted**		Model 3: Multivariable-adjusted* + adjustment for competing risk of AF	
		HR (95% CI)	*P*-value	HR (95% CI)	*P*-value	sHR (95% CI)	*P*-value
**SNI score**							
0—Low	28/81	1.90 (1.26–2.87)	0.002	1.57 (1.03–2.39)	0.04	1.50 (0.98–2.31)	0.06
1—Medium–Low	175/525	1.46 (1.16–1.84)	0.001	1.31 (1.04–1.65)	0.02	1.42 (1.12–1.81)	0.004
2—Medium	334/1119	1.27 (1.03–1.55)	0.02	1.19 (0.97–1.46)	0.09	1.26 (1.03–1.55)	0.02
3—Medium–High	292/1079	1.10 (0.90–1.35)	0.36	1.09 (0.89–1.34)	0.42	1.16 (0.94–1.43)	0.16
4—High	136/650	1.00 (referent)	–	1.00 (referent)	–	1.00 (referent)	–
TOTAL	965/3454	*Overall p-value*	0.0006	*Overall p-value*	0.06	*Overall p-value*	0.03
**SNI components**							
**Currently married**							
Yes	556/2338	0.96 (0.83–1.11)	0.59	1.01 (0.87–1.18)	0.87	1.07 (0.92–1.25)	0.35
No	409/1116	1.00 (referent)	–	1.00 (referent)	–	1.00 (referent)	–
**Number of close friends and relatives**							
≥ 3 friends and ≥ relatives	778/2815	0.95 (0.81–1.12)	0.54	0.99 (0.84–1.16)	0.86	0.97 (0.82–1.15)	0.72
0–2 friends and 0–2 relatives	187/639	1.00 (referent)	–	1.00 (referent)	–	1.00 (referent)	–
**Frequency of religious service attendance**							
≥ 1 time per month	495/1802	0.71 (0.62–0.81)	< 0.0001	0.74 (0.65–0.85)	< 0.0001	0.76 (0.67–0.87)	< 0.0001
< 1 time per month	470/1652	1.00 (referent)	–	1.00 (referent)	–	1.00 (referent)	–
**Social group participation**							
Yes	434/1645	0.85 (0.75–0.97)	0.02	0.91 (0.80–1.03)	0.15	0.81 (0.71–0.93)	0.002
No	531/1809	1.00 (referent)	–	1.00 (referent)	–	1.00 (referent)	–

SNI, social network index; AF, atrial fibrillation; HR, cause-specific hazards ratio; sHR, subdistribution hazards ratio; CI, confidence interval.

All models are stratified by cohort membership and adjusted for time between SNI measurement and covariate measurement. Multiple imputation was implemented to handle missing covariate data.

*There were 1373 total deaths during the follow-up period. There were 965 mortality without AF diagnosis events (deaths occurring among individuals who did not develop AF during follow-up).

**Adjusted for age, sex, height, weight, systolic blood pressure, diastolic blood pressure, hypertension treatment, current smoking, diabetes, history of myocardial infarction, and history of heart failure. Participants censored at AF diagnosis.

Religious service attendance was inversely associated with mortality without AF diagnosis in all models (sHR = 0.76, 95% CI 0.67–0.87, *p*-value < 0.0001). Social group participation was inversely associated with mortality without AF diagnosis in the age- and sex- adjusted Cox model (HR = 0.85, 95% CI 0.75–0.97, *p*-value = 0.02) and in the multivariable-adjusted Fine-Gray subdistribution hazards model (sHR = 0.81, 95% CI 0.71–0.93, *p*-value = 0.002). Marital status and number of friends and relatives were not significantly associated with mortality without AF diagnosis in any of the models.

### Association between social network index and all-cause mortality

Low SNI score was associated with a higher rate of all-cause mortality in age- and sex-adjusted Cox model (*p*-value = 0.0009) and in multivariable Cox model (*p*-value = 0.03; Supplemental Table S7). As compared to the highest SNI group, participants in the lowest SNI group had a 62% higher rate of dying (*p*-value = 0.007) after multivariable-adjustment. Of the four SNI components, only religious service attendance showed a statistically significant association with all-cause mortality risk; religious service attendees had a 19% lower rate of death (*p*-value = 0.0003).

## Discussion

### Principal findings

Examining Framingham Heart Study data, we analyzed the association between social integration and incident AF, mortality without AF diagnosis, and all-cause death. We observed that social isolation was associated with a lower incidence of AF but also a higher incidence of mortality without AF diagnosis. The lower incidence of AF was primarily associated with a single component of the SNI score, social group participation. Individuals who participated in social groups had a higher incidence of AF as compared to non-participants. Both social group participation and regular religious service attendance were associated with a higher incidence of mortality without AF diagnosis. Finally, no association was found for marital status or for number of close friends/relatives for any of the outcomes studied.

Our findings were contrary to our hypothesis that lower SNI scores would be associated with increased incident AF rates. There are several potential explanations of our findings. Our observations potentially may be explained by the competing risk of mortality. In fact, lower SNI scores were associated with increased mortality rates. Because participants more socially isolated are at high risk for death before experiencing AF and there were many more deaths (*N* = 1373 for all-cause mortality) as compared to incident AF events (*N* = 686) during the study period; it appears that lower SNI scores may be inversely associated with AF. In addition, it is possible that individuals with lower social connections may have less access to medical care and therefore may have been less likely to have their AF diagnosed. Previous studies of various outcomes in the presence of competing risk of mortality have also noted a reversal of the expected direction of association if there is a strong association between the exposure of interest and mortality^23,24^.

### Comparison with previous studies

Importance of social network on individual behavior for BMI changes^25^ and particularly obesity, which is a known risk factor associated with AF, has already been reported in the Framingham Heart Study. Also, social isolation and lacking social support are associated with cardio- and cerebrovascular outcomes related to AF. It has been reported that social support is an important predictor of recovery after stroke^26^ and myocardial infarction^10^. Furthermore, as demonstrated in a large Danish population cohort, social integration predicted incidence of cardiovascular disease and mortality^3–5^. Interestingly, when analyzing social factors related to adverse outcomes, not having a spouse or a partner was associated with the highest mortality rate as compared to being married or in a partnership^3^. This finding was in accordance with previous results reported by Berkman and colleagues demonstrating that married individuals have lower mortality rates compared to non-married^1^. Furthermore, the authors reported relevant sex-associated differences, with higher risk in men.

The original study of social networks and mortality among Alameda County residents by Berkman and colleagues demonstrated that each of the four components of the SNI was associated with mortality risk. In our study we did not confirm these results. However, their measurement of SNI occurred in 1965, 30 + years prior to our study’s baseline visit, and it is unclear how the importance of various social connections may have changed over time. In our study, we observed that among four SNI components (marital status, close friends/relatives, social group participation, and regular religious service attendance), only social group participation was associated with incident AF. Furthermore, together with regular religious service attendance, social group participation was associated with reduced mortality without AF diagnosis. However, an association was not observed between marital status and either incident AF or with mortality.

A recent analysis from the Atherosclerosis Risk in Communities Study reported no evidence of an association between deficient social ties and AF incidence while an association with vital exhaustion had been reported^27^. One of the main strengths of the latter study is analysis in > 11,000 bi-racial participants with > 2220 incident AF cases during 23 years follow-up. In their study, Garg et al. examined social connectedness using Interpersonal Support Evaluation List and the Lubben Social Network Scale^28,29^ in contrast to our study, which used the Berkman-Syme SNI index. Also, Garg et al. combined two lowest social connectedness groups because of limited participants’ number, while we considered all SNI score groups separately.

### Biological plausibility

Social isolation is an important risk factor for unhealthy behavior including inactive lifestyle and alcohol consumption, which are known risk factors for cardiovascular diseases^30,31^ and AF. Social integration, which describes the presence of close personal ties to family and friends, and social ties to community, has been reported to predict CVD incidence and mortality in several prospective studies^5^. Primary studies have highlighted three pathways through which social relationships can influence CVD risk: behavioral (e.g., smoking, physical inactivity); psychological (e.g., low self-esteem and self-efficacy); and biological (e.g., response to stress, psychological load, and cardiovascular reactivity)^32^. A meta-analysis found that weaker social relationships were associated with a ~ 30% increase in the risk of incident heart disease and similar increase in the risk of stroke^15^. Chang et al.^33^ demonstrated that social integration was inversely associated with CVD incidence in women.

Poor social support may lead to increased stress and partly explain unhealthy behavior^13,34^. One of the risk factors linking AF with SNI might be psychological stress. Pathophysiologically, psychological stress activates the autonomic nervous system, hypothalamus–pituitary–adrenal axis, and renin–angiotensin–aldosterone system^35^, which play crucial roles in AF initiation due to underlying pro-fibrotic changes^36^. Also, stress might indirectly provoke atrial electrophysiological remodeling accelerating AF development^37^. Nevertheless, studies analyzing the effect of chronic stress, depression, hostility, anger, and tension on AF incidence reported mixed results^38,39^. There is evidence that psychosocial stress and associated negative emotions were the most common triggering factors for AF paroxysms^9^. While positive emotions were not linked to AF paroxysms, negative emotions lead to hormonal dysregulation and inflammation facilitating AF initiation trough atrial fibrosis^35^.

Individuals with better social support have more resources to help maintain physical health and have higher health literacy regarding preventive and health-promoting procedures^40^. Additionally, increased social support may help an individual better cope with stressful events, reducing the adverse physiological effects associated with stress. Also, analyzing biological effects of social isolation on health, Cole et al. found differences in gene expression among individuals with different social support^41^. Thus, individuals with poor social support had higher expression of genes association with pro-inflammatory signaling, while the expression of genes associated with antiviral resistance, antibody production, and white blood cells function was lower.

In our analysis, we did not observe that social isolation was associated with a higher risk for incident AF. However, we found that individuals with lower SNI had a higher incidence of mortality without AF diagnosis suggesting competing nature of social isolation and mortality. Also, our findings might be explained by differences in AF awareness, diagnosis, health literacy, availability of care, and treatment access in individuals with lower SNI (e.g., social isolation). For instance, socially isolated individuals may access follow up care less frequently than their socially connected peers.

The World Health Organization and European Union initiatives recognized importance of social connectedness in supporting health status. This had led to the development of “healthy ageing” campaigns and organization of “age-friendly” cities tackling social connectedness as one of the wellbeing factors.

In a study analyzing mortality risk, the effects of social isolation were comparable to that of “classical” risk factors such as high blood pressure and smoking^5^. Assessing an individual’s social connectedness would complement addressing unhealthy behaviors (e.g., smoking, alcohol consume, physical inactivity)^30,31^ and also other risk factors impairing cardiovascular health (e.g., depression, anxiety)^42^. Although it is possible that assessment of social connectedness might be impaired by individual’s reluctance to talk about negative feelings or social ties publicly, indirect assessments (e.g., the de Jong Gierveld loneliness^43^ or UCLA scales^44^) may be helpful in addressing such challenges.

## Limitations

There are several limitations that should be considered when interpreting the current findings. First, the Framingham Heart Study is an observational study and therefore residual confounding cannot be ruled out and the study cannot establish causal relations. Our analysis did not include variables such as alcohol consumption, obstructive sleep apnea, or physical activity, which may affect the likelihood of AF development. Secondly, the current study included individuals aged ≥ 55 years, largely of European ancestry, and living in New England. Although the analysis included participants from underrepresented racial and ethnicity groups, the number of individuals from the Framingham Heart Study Omni 1 cohort comprised only a small proportion of the study sample. Therefore, the generalizability to younger ages, other races and ethnicities, and other regions/countries is unknown and should be addressed in other epidemiological cohorts. Thirdly, since AF may be undiagnosed, there may have been misclassification of occurrence and timing of AF onset. Furthermore, there may be misclassification of social networks. The SNI score was measured several years prior to AF onset and we were not able to analyze changes in social connectedness over time. Additionally, we were unable to account for recent changes in social connectedness, such as social media, that have occurred years since the SNI measurement was performed. Finally, the number of participants who reported no social connections (SNI score of zero) was extremely low, so analyses of the SNI score 0 group were underpowered. We observed a nonlinear association of SNI to AF, which is probably a reflection of competing risk of AF-free mortality and ascertainment biases of SNI = 0 participants accessing clinical care less. Therefore, our results should be viewed as hypothesis generating.

## Conclusions

Poor social connectedness, as measured by the SNI, was associated with a higher risk of mortality. In contrast to our hypothesis, we observed a seemingly protective association of social isolation with AF incidence. This finding should be interpreted with caution since it is likely an artifact of the strong association between social isolation and increased mortality rate in combination with the large number of deaths as compared to AF events. Interestingly, among four SNI components, only social group participation was associated with increased incident AF, while social group participation and regular religious service attendance were associated with a decreased rate of mortality without AF diagnosis.

## Supplementary Information

Supplementary Information.

## Data Availability

The authors confirm that the data supporting the findings of this study are available within the article and its supplementary materials.
