# Supplementary material for: The association between social network index, atrial fibrillation, and mortality in the Framingham Heart Study
Source: Sci Rep. 2022 Mar 10;12:3958. doi: 10.1038/s41598-022-07850-9 (PMC8913787; doi:10.1038/s41598-022-07850-9)
Supplement: Supplementary file 1 — Supplementary Information. [file 41598_2022_7850_MOESM1_ESM.docx]

**The association between social network index, atrial fibrillation, and mortality**

**in the Framingham Heart Study**

**Table S1**. Study sample characteristics for Framingham Heart Study Original cohort participants.

**Table S2**. Study sample characteristics for Framingham Heart Study Offspring cohort participants.

**Table S3**. Study sample characteristics for Framingham Heart Study OMNI 1 cohort participants.

**Table S4**. Summary of missing values in the study sample.

**Table S5**. Summary statistics comparing those with missing covariates to complete cases.

**Table S6**. Hazards ratios for the association between social network index and incident **atrial fibrillation** (Original, Offspring, Omni1) using complete case analysis sample.

**Table S7.** Hazard ratios for the association between social network index and **all-cause death** in the Framingham Heart Study Original, Offspring, and Omni 1 cohorts.

**Supplemental Table 1**. Study sample characteristics for Framingham Heart Study Original cohort participants (N=537).

|  | **Social Network Index Score** | | | | | **Total**  **(N=537)** |
| --- | --- | --- | --- | --- | --- | --- |
|  | **Low (0)**  **(N=21)** | **Medium-Low (1)**  **(N=109)** | **Medium (2)**  **(N=183)** | **Medium-High (3)**  **(N=168)** | **High (4)**  **(N=56)** |  |
| Age, years | 85.0±5.0 | 85.6±4.8 | 84.0±4.0 | 83.5±4.0 | 82.2±2.3 | 84.0±4.2 |
| Female sex | 19 (90.5) | 77 (70.6) | 132 (72.1) | 123 (73.2) | 29 (51.8) | 380 (70.8) |
| Years of follow-up for incident AF* | 4.4±2.9 | 6.6±4.7 | 6.8±4.8 | 7.8±4.8 | 7.5±4.7 | 7.0±4.7 |
| Systolic blood pressure, mm Hg | 139±20 | 143±23 | 142±19 | 141±21 | 139±17 | 141±20 |
| Diastolic blood pressure, mm Hg | 71±13 | 70±12 | 70±11 | 68±11 | 69±12 | 69±11 |
| Height, inches | 61±3 | 63±4 | 62±4 | 62±4 | 64±4 | 63±4 |
| Weight, pounds | 135±36 | 143±32 | 147±31 | 146±28 | 155±33 | 147±31 |
| Hypertension treatment | 11 (52.4) | 63 (58.3) | 107 (59.4) | 96 (57.8) | 30 (53.6) | 307 (57.8) |
| Current smoker | 2 (9.5) | 10 (9.3) | 12 (6.6) | 6 (3.6) | 2 (3.6) | 32 (6.0) |
| Diabetes | ** | ** | ** | ** | ** | ** |
| History of CHF | 1 (4.8) | 7 (6.4) | 8 (4.4) | 6 (3.6) | 0 (0.0) | 22 (4.1) |
| History of MI | 0 (0.0) | 6 (5.5) | 11 (6.0) | 16 (9.5) | 4 (7.1) | 37 (6.9) |
| Social Network Index components |  |  |  |  |  |  |
| Currently married | 0 (0.0) | 16 (14.7) | 59 (32.2) | 64 (38.1) | 56 (100.0) | 195 (36.3) |
| >2 close friends and >2 close relatives | 0 (0.0) | 69 (63.3) | 146 (80.0) | 154 (92.7) | 56 (100.0) | 425 (79.1) |
| Regular religious service attendance | 0 (0.0) | 12 (11.0) | 83 (45.4) | 152 (90.5) | 56 (100.0) | 303 (45.4) |
| Participates in social group | 0 (0.0) | 12 (11.0) | 78 (42.6) | 134 (79.8) | 56 (100.0) | 280 (52.1) |

Note: Tables values represent mean±SD or n (%).

* Note that the follow-up time represents the total time the participants were under surveillance for outcome events by FHS research staff. Regardless of their study visit attendance, all FHS participants are routinely monitored via mailed questionnaire and/or telephone contact until their death or withdrawal from the study. The median (25^th^, 75^th^ percentile) number of follow-up visits attended after the study visit where SNI was assessed was 2 (1, 4). The maximum possible visits for the Original cohort prior to the end of follow up (12/31/2016) was 7.

**Diabetes status is missing for the majority of the Original cohort participants since fasting blood glucose was not measured during later exams.

**Supplemental Table 2**. Study sample characteristics for Framingham Heart Study Offspring cohort participants (N=2684).

|  | **Social Network Index Score** | | | | | **Total**  **(N=2684)** |
| --- | --- | --- | --- | --- | --- | --- |
|  | **Low (0)**  **(N=60)** | **Medium-Low (1)**  **(N=397)** | **Medium (2)**  **(N=885)** | **Medium-High (3)**  **(N=826)** | **High (4)**  **(N=516)** |  |
| Age, years | 63.0±7.8 | 63.6±7.0 | 64.2±7.3 | 64.9±7.1 | 65.4±6.9 | 64.5±7.1 |
| Female sex | 35 (58.3) | 211 (53.2) | 447 (50.5) | 491 (59.4) | 297 (57.6) | 1481 (55.2) |
| Years of follow-up for incident AF* | 11.9±4.3 | 12.3±5.1 | 12.6±4.8 | 12.9±4.6 | 12.9±4.8 | 12.7±4.8 |
| Systolic blood pressure, mm Hg | 127±18 | 128±18 | 131±19 | 129±18 | 130±18 | 130±19 |
| Diastolic blood pressure, mm Hg | 74±9 | 74±10 | 75±10 | 73±10 | 74±10 | 74±10 |
| Height, inches | 66±4 | 66±4 | 66±4 | 65±4 | 66±4 | 66±4 |
| Weight, pounds | 183±49 | 173±39 | 175±37 | 173±38 | 172±37 | 173±38 |
| Hypertension treatment | 22 (37.3) | 168 (42.4) | 354 (40.1) | 310 (37.5) | 205 (39.7) | 1059 (39.5) |
| Current smoker | 23 (38.3) | 77 (19.4) | 111 (12.5) | 71 (8.6) | 25 (4.8) | 307 (11.4) |
| Diabetes | 8 (14.6) | 42 (11.3) | 115 (13.6) | 96 (12.2) | 59 (11.9) | 320 (12.5) |
| History of CHF | 0 (0.0) | 6 (1.5) | 3 (0.3) | 4 (0.5) | 3 (0.6) | 16 (0.6) |
| History of MI | 2 (3.3) | 17 (4.3) | 39 (4.4) | 17 (2.1) | 12 (2.3) | 87 (3.2) |
| Social Network Index components |  |  |  |  |  |  |
| Currently married | 0 (0.0) | 160 (40.3) | 650 (73.4) | 641 (77.6) | 516 (100.0) | 1967 (73.3) |
| >2 close friends and >2 close relatives | 0 (0.0) | 198 (49.9) | 733 (82.8) | 758 (91.8) | 516 (100.0) | 2205 (82.2) |
| Regular religious service attendance | 0 (0.0) | 21 (5.3) | 214 (24.2) | 593 (71.8) | 516 (100.0) | 1344 (50.1) |
| Participates in social group | 0 (0.0) | 18 (4.5) | 173 (19.6) | 486 (58.8) | 516 (100.0) | 1193 (44.5) |

Note: Tables values represent mean±SD or n (%).

* Note that the follow-up time represents the total time the participants were under surveillance for outcome events by FHS research staff. Regardless of their study visit attendance, all FHS participants are routinely monitored via mailed questionnaire and/or telephone contact until their death or withdrawal from the study. The median (25^th^, 75^th^ percentile) number of follow-up visits attended after the study visit where SNI was assessed was 2 (1, 2). The maximum possible visits for the Offspring prior to the end of follow up (12/31/2016) was 2.

**Supplemental Table 3**. Study sample characteristics for Framingham Heart Study OMNI 1 cohort participants (N=233).

|  | **Social Network Index Score** | | | | | **Total**  **(N=233)** |
| --- | --- | --- | --- | --- | --- | --- |
|  | **Low (0)**  **(N=0)** | **Medium-Low (1)**  **(N=19)** | **Medium (2)**  **(N=51)** | **Medium-High (3)**  **(N=85)** | **High (4)**  **(N=78)** |  |
| Age, years | --- | 61.3±4.2 | 61.0±6.0 | 62.7±6.6 | 62.1±5.9 | 62.0±6.1 |
| Female sex | 0 (0.0) | 9 (47.4) | 26 (51.0) | 56 (65.9) | 41 (52.6) | 132 (56.7) |
| Years of follow-up for incident AF | --- | 13.2±4.8 | 12.2±4.2 | 12.8±4.4 | 12.8±4.3 | 12.7±4.3 |
| Systolic blood pressure, mm Hg | --- | 134±16 | 129±13 | 135±19 | 130±18 | 132±17 |
| Diastolic blood pressure, mm Hg | --- | 80±9 | 78±8 | 77±10 | 77±10 | 77±10 |
| Height, inches | --- | 65±4 | 65±4 | 64±4 | 65±4 | 65±4 |
| Weight, pounds | --- | 169±36 | 167±69 | 168±40 | 171±39 | 169±39 |
| Hypertension treatment | 0 (0.0) | 3 (15.8) | 26 (51.0) | 33 (39.8) | 26 (33.8) | 88 (38.3) |
| Current smoker | 0 (0.0) | 4 (21.1) | 3 (5.9) | 7 (8.2) | 3 (3.9) | 17 (7.3) |
| Diabetes | 0 (0.0) | 2 (10.5) | 6 (12.0) | 11 (12.9) | 12 (15.4) | 31 (13.4) |
| History of CHF | 0 (0.0) | 0 (0.0) | 1 (2.0) | 0 (0.0) | 2 (2.6) | 3 (1.3) |
| History of MI | 0 (0.0) | 0 (0.0) | 2 (3.9) | 1 (1.2) | 3 (3.9) | 6 (2.6) |
| Social Network Index components |  |  |  |  |  |  |
| Currently married | 0 (0.0) | 14 (73.7) | 34 (66.7) | 50 (58.8) | 78 (100.0) | 176 (75.5) |
| >2 close friends and >2 close relatives | 0 (0.0) | 5 (26.3) | 33 (64.7) | 69 (81.2) | 78 (100.0) | 185 (79.4) |
| Regular religious service attendance | 0 (0.0) | 0 (0.0) | 15 (29.4) | 62 (72.9) | 78 (100.0) | 155 (66.5) |
| Participates in social group | 0 (0.0) | 0 (0.0) | 20 (39.2) | 74 (87.1) | 78 (100.0) | 172 (73.8) |

Note: Tables values represent mean±SD or n (%).

***** Note that the follow-up time represents the total time the participants were under surveillance for outcome events by FHS research staff. Regardless of their study visit attendance, all FHS participants are routinely monitored via mailed questionnaire and/or telephone contact until their death or withdrawal from the study. The median (25^th^, 75^th^ percentile) number of follow-up visits attended after the study visit where SNI was assessed was 1 (1, 2). The maximum possible visits for the OMNI 1 prior to the end of follow up (12/31/2016) was 2.

**Table S4**. Summary of missing values in the study sample (N=3454).

| **Variable** | **N missing** |
| --- | --- |
| Age | 0 |
| SBP | 0 |
| DBP | 3 |
| Height | 83 |
| Weight | 33 |
| Female sex | 0 |
| Current smoking | 2 |
| Diabetes | 650 |
| History of MI | 0 |
| History of CHF | 0 |
| Hypertension treatment | 13 |

**Table S5**. Summary statistics comparing those with missing covariates to complete cases.

| **Variable** | **Complete**  **Cases**  **(N=2789)** | **Missing 1+ Covariates (N=665)** | **P-value** |
| --- | --- | --- | --- |
| Age, median (25^th^, 75^th^ percentile) | 62 (58, 69) | 82 (79, 86) | <0.0001 |
| Systolic blood pressure | 130 (18) | 140 (21) | <0.0001 |
| Female sex | 1536 (55.1) | 457 (68.7) | <0.0001 |
| FHS Cohort |  |  |  |
| Original | 13 (0.5) | 524 (78.8) | <0.0001 |
| Offspring | 2548 (91.4) | 136 (20.5) |  |
| Omni 1 | 228 (8.2) | 5 (0.8) |  |
| History of MI | 84 (3.0) | 46 (6.9) | <0.0001 |
| History of CHF | 17 (0.6) | 24 (3.6) | <0.0001 |
| Social network index score |  |  |  |
| 0 | 56 (2.0) | 25 (3.8) | <0.0001 |
| 1 | 392 (14.1) | 133 (20.0) |  |
| 2 | 898 (32.2) | 221 (33.2) |  |
| 3 | 870 (31.2) | 209 (31.4) |  |
| 4 | 573 (20.5) | 77 (11.6) |  |
| Social Network Index components |  |  |  |
| Currently married | 2059 (73.8) | 279 (42.0) | <0.0001 |
| >2 close friends and >2 close relatives | 2281 (81.8) | 534 (80.3) | 0.38 |
| Participates in social group | 1305 (46.8) | 340 (51.1) | 0.04 |
| Regular religious service attendance | 1445 (51.8) | 357 (53.7) | 0.38 |

Note: Table values are mean (SD) or n (%) unless otherwise indicated.

**Table S6.** Hazards ratios for the association between social network index and incident **atrial fibrillation** (Original, Offspring, Omni1) using complete case analysis sample.

| **Outcome** | **Social Network**  **Index Score** | **# AF cases/**  **# Participants** | **Model 1: Age/sex adjusted** | | **Model 2: Multivariable-adjusted*** | | **Model 3: Multivariable-adjusted* + Adjusted for competing risk of mortality** | |  |
| --- | --- | --- | --- | --- | --- | --- | --- | --- | --- |
|  |  |  | **HR (95% CI)** | **P-value** | **HR (95% CI)** | **P-value** | **sHR (95% CI)** | **P-value** |  |
| Atrial Fibrillation | 0 - Low | 15/56 | 1.64 (0.96-2.82) | 0.07 | 1.64 (0.94-2.85) | 0.08 | 1.61 (0.96-2.70) | 0.07 |  |
|  | 1 - Medium-Low | 54/392 | 0.82 (0.59-1.14) | 0.24 | 0.81 (0.58-1.13) | 0.22 | 0.72 (0.51-1.00) | 0.05 |  |
|  | 2 - Medium | 165/898 | 0.97 (0.76-1.24) | 0.80 | 0.96 (0.75-1.22) | 0.72 | 0.92 (0.72-1.18) | 0.51 |  |
|  | 3 - Medium-High | 159/870 | 0.99 (0.78-1.27) | 0.94 | 1.01 (0.79-1.29) | 0.93 | 0.97 (0.76-1.25) | 0.83 |  |
|  | 4 - High | 109/573 | 1.00 (referent) | --- | 1.00 (referent) | --- | 1.00 (referent) | --- |  |
|  | TOTAL | 502/2789 | *Overall p-value* | 0.41 | *Overall p-value* | 0.20 | *Overall p-value* | 0.05 |  |
|  | | | | | | | | | |
| AF-free Mortality | 0 - Low | 10/56 | 1.33 (0.69-2.55) | 0.39 | 0.91 (0.47-1.78) | 0.79 | 0.83 (0.40-1.70) | 0.61 |  |
|  | 1 - Medium-Low | 84/392 | 1.52 (1.13-2.04) | 0.005 | 1.34 (0.99-1.80) | 0.06 | 1.37 (1.01-1.86) | 0.04 |  |
|  | 2 - Medium | 192/898 | 1.27 (1.00-1.62) | 0.05 | 1.15 (0.90-1.47) | 0.27 | 1.17 (0.91-1.50) | 0.21 |  |
|  | 3 - Medium-High | 172/870 | 1.16 (0.91-1.49) | 0.24 | 1.15 (0.90-1.48) | 0.27 | 1.14 (0.89-1.47) | 0.30 |  |
|  | 4 - High | 99/573 | 1.00 (referent) | --- | 1.00 (referent) | --- | 1.00 (referent) | --- |  |
|  | TOTAL | 557/2789 | *Overall p-value* | 0.07 | *Overall p-value* | 0.38 | *Overall p-value* | 0.27 |  |
|  | | | | | | | | | |
| Death | 0 - Low | 17/56 | 1.32 (0.80-2.18) | 0.28 | 1.10 (0.66-1.83) | 0.73 | --- | --- |  |
|  | 1 - Medium-Low | 114/392 | 1.44 (1.13-1.84) | 0.004 | 1.33 (1.04-1.70) | 0.02 | *---* | --- |  |
|  | 2 - Medium | 270/898 | 1.20 (0.98-1.46) | 0.07 | 1.13 (0.92-1.38) | 0.23 | *---* | --- |  |
|  | 3 - Medium-High | 246/870 | 1.12 (0.91-1.37) | 0.29 | 1.12 (0.91-1.37) | 0.29 | *---* | --- |  |
|  | 4 - High | 153/573 | 1.00 (referent) | --- | 1.00 (referent) | --- | *---* | --- |  |
|  | TOTAL | 800/2789 | *Overall p-value* | 0.05 | *Overall p-value* | 0.27 | *---* | --- |  |
| Abbreviations: SNI, social network index; AF, atrial fibrillation; HR, cause-specific hazards ratio; sHR, subdistribution hazards ratio; CI, confidence interval  Note: All models are stratified by cohort membership and adjusted for time between SNI measurement and covariate measurement.  *Adjusted for age, sex, height, weight, systolic blood pressure, diastolic blood pressure, hypertension treatment, current smoking, diabetes, history of MI, and history of CHF. | | | | | | | | | |

| **Table S7.** Hazard ratios for the association between social network index and **all-cause death** in the Framingham Heart Study Original, Offspring, and Omni 1 cohorts. | | | | | | |
| --- | --- | --- | --- | --- | --- | --- |
| **Social Network**  **Index** | **# Deaths/**  **# Participants** | **Model 1: Age/sex adjusted** | | **Model 2: Multivariable-adjusted*** | |  |
|  |  | **HR (95% CI)** | **P-value** | **HR (95% CI)** | **P-value** |  |
| *SNI score* |  |  |  |  |  |  |
| 0 - Low | 39/81 | 1.79 (1.27-2.53) | 0.0009 | 1.62 (1.14-2.29) | 0.007 |  |
| 1 - Medium-Low | 231/525 | 1.35 (1.12-1.63) | 0.002 | 1.26 (1.04-1.52) | 0.02 |  |
| 2 - Medium | 466/1119 | 1.21 (1.03-1.43) | 0.02 | 1.15 (0.97-1.35) | 0.10 |  |
| 3 - Medium-High | 421/1079 | 1.11 (0.94-1.30) | 0.24 | 1.08 (0.92-1.28) | 0.36 |  |
| 4 - High | 216/650 | 1.00 (referent) | --- | 1.00 (referent) | --- |  |
| TOTAL | 1373/3454 | *Overall p-value* | 0.0009 | *Overall p-value* | 0.03 |  |
| *SNI components* |  |  |  |  |  |  |
| Currently married |  |  |  |  |  |  |
| Yes | 792/2338 | 0.91 (0.81-1.03) | 0.14 | 0.95 (0.84-1.08) | 0.44 |  |
| No | 581/1116 | 1.00 (referent) | --- | 1.00 (referent) | --- |  |
| Number of close friends and relatives |  |  |  |  |  |  |
| ≥3 friends and ≥relatives | 1101/2815 | 0.93 (0.81-1.06) | 0.26 | 0.95 (0.83-1.08) | 0.41 |  |
| 0-2 friends and 0-2 relatives | 272/639 | 1.00 (referent) | --- | 1.00 (referent) | --- |  |
| Frequency of religious service attendance |  |  |  |  |  |  |
| ≥1 time per month | 742/1802 | 0.79 (0.71-0.88) | <0.0001 | 0.81 (0.73-0.91) | 0.0003 |  |
| <1 time per month | 631/1652 | 1.00 (referent) | --- | 1.00 (referent) | --- |  |
| Social group participation |  |  |  |  |  |  |
| Yes | 655/1645 | 0.90 (0.81-1.00) | 0.06 | 0.93 (0.84-1.04) | 0.22 |  |
| No | 718/1809 | 1.00 (referent) | --- | 1.00 (referent) | --- |  |
| Abbreviations: SNI, social network index; AF, atrial fibrillation; HR, cause-specific hazards ratio; CI, confidence interval  Note: All models are stratified by cohort membership and adjusted for time between SNI measurement and covariate measurement. Multiple imputation was implemented to handle missing covariate data.  *Adjusted for age, sex, height, weight, systolic blood pressure, diastolic blood pressure, hypertension treatment, current smoking, diabetes, history of MI, and history of CHF. | | | | | | |
